# Matrilin-2 Is Proteolytically Cleaved by ADAMTS-4 and ADAMTS-5

**DOI:** 10.3390/molecules19068472

**Published:** 2014-06-23

**Authors:** Zhengke Wang, Junming Luo, Satori Iwamoto, Qian Chen

**Affiliations:** 1 Cell and Molecular Biology Laboratory, Department of Orthopaedics, The Warren Alpert Medical School of Brown University, Rhode Island Hospital, Providence, RI 02903, USA; 2 Department of Dermatology, Roger Williams Medical Center, Providence, RI 02908, USA

**Keywords:** matrilin-2, proteolytic cleavage, ADAMTS-4, ADAMTS-5, oligomerization

## Abstract

Matrilin-2 is a widely distributed, oligomeric extracellular matrix protein that forms a filamentous network by binding to a variety of different extracellular matrix proteins. We found matrilin-2 proteolytic products in transfected cell lines *in vitro* and in mouse tissues *in vivo*. Two putative cleavage sites were identified in the unique domain of matrilin-2; the first site was located between D^851^ and L^852^ in the middle of the domain and the second, at the boundary with the coiled-coil domain at the C-terminus. Deletion of the entire unique domain eliminated the proteolysis of matrilin-2. While the first cleavage site was present in all matrilin-2 oligomers, the second cleavage site became apparent only in the matrilin-2 hetero-oligomers with matrilin-1 or matrilin-3. Analysis using a variety of extracellular protease inhibitors suggested that this proteolytic activity was derived from a member or several members of the ADAMTS family. Recombinant human ADAMTS-4 (aggrecanase-1) and ADAMTS-5 (aggrecanase-2), but not ADAMTS-1, cleaved recombinant matrilin-2, thereby yielding matrilin-2 proteolytic peptides at the predicted sizes. These results suggest that ADAMTS-4 and ADAMTS-5 may destabilize the filamentous network in the extracellular matrix by cleaving matrilin-2 in both homo-oligomers and hetero-oligomers.

## 1. Introduction

Matrilins are a family of four non-collagenous extracellular matrix (ECM) proteins [1,2]. Among them, matrilin-1 [3] and matrilin-3 [4,5] are expressed mainly in cartilage, while matrilin-2 [6] and matrilin-4 [7,8] are widely distributed in many connective tissues [9]. Matrilin-2 (MATN2) is the largest and the most complex matrilin. It is made up by two vWFA-like domains flanked by ten EGF modules with a unique domain located between the vWFA2 and the coiled-coil domain at its C-terminus [6]. Matrilin-2 is widely distributed in extracellular matrices of many connective tissues [9,10]. Matrilin-2 can bind to collagen I and non-collagenous proteins, such as fibrillin-2, fibronectin, and laminin-1-nidogen-1 complexes [11]. Matrilin-2 is expressed by pre-myelinating Schwann cells during normal development. It increases neurite outgrowth of dorsal root ganglia (DRG) neurons and enhances the migration of embryonic DRG-derived Schwann cells [12]. In addition, matrilin-2 is synthesized by both keratinocytes and dermal fibroblasts in human skin, which is further processed by cell-associated proteases [13]. Matrilin-2 gene expression is regulated by BMP-7 and the transcription factor DeltaNp63, which is responsible for keratinocyte cell migration [14].

Extracellular proteinases are required for numerous developmental and homeostasis processes. Excessive degradation or accumulation of extracellular matrix macromolecules, as a result of malfunction of extracellular proteinases or their matrix substrates, leads to various human diseases. For example, degeneration and loss of extracellular matrix macromolecules from cartilage result in serious impairment of joint function. Extracellular proteinases include both matrix metalloproteinases (MMP), which are extensively characterized, and the ADAMTS family members. The ADAMTS proteases are extracellular matrix proteins with ADAM-like protease domain and matrix-binding thrombospondin type 1-repeat [15,16]. Among them, two cartilage aggrecanases, aggrecanase-1 (ADAMTS-4) and aggrecanase-2 (ADAMTS-5) are upregulated in human osteoarthritic (OA) cartilage. They are responsible for aggrecan degradation in the absence of other matrix metalloproteinases [17]. ADAMTS-4 is a glutamyl endopeptidase that preferentially cleaves Glu-Xaa bonds of the core protein of proteoglycans such as aggrecan, brevican, and versican [18,19]. This proteolytic process was thought to depend on the presence of glycosaminoglycans in the substrate [20,21].

Matrilins form a filamentous network to connect their ligands (including integrins [22], collagens [23,24], proteoglycans [25], and other non-collagenous glycoproteins) in the extracellular matrix [26]. All matrilins contain the following: one or two von Willebrand Factor (vWF) A domains, a variable number of epidermal growth factor (EGF) domains, and a coiled-coil domain at the *C*-terminal end [1]. The vWF A domain is responsible for the interaction of matrilins with their matrix ligands through a metal-ion dependent adhesion site [3]. The coiled-coil domain is responsible for oligomerization of matrilin subunits, which include homo-oligomerization, or hetero-oligomerization if two different matrilin molecules are expressed in the same tissue at the same time [27,28]. We previously found evidence for a proteolytic cleavage site between the vWF domain and the coiled-coil domain in matrilin-1 [29]. Proteolytic cleavage may decrease matrilins’ ability to bind other extracellular matrix proteins when the hetero-oligomers are proteolyzed into fragments. It was recently shown that matrilin-3 [30] and matrilin-4 [31] are both cleaved by ADAMTS-4 in the hinge region at the specific Glu-Xaa bond. Physiological cleavage has been described for most members of the matrilin family members. Interestingly, an identified cleavage of matrilin-4 [31] at the N-terminus of the coiled-coil domain, is conserved throughout the matrilin family [29,30]. Our previous work had shown that two alternatively-spliced isoforms in the unique domain of matrilin-2 resulted in different matrilin-2 oligomers [32]. In this study, we analyzed matrilin-2 proteolytic processing and found matrilin-2 to be a substrate of aggrecanase-1 (ADAMTS-4) and aggrecanase-2 (ADAMTS-5). Two separate cleavage sites were identified in matrilin-2. One was located at the boundary of the unique domain with the coiled-coil domain as in other matrilin cleavage sites. The other cleavage site was a previously unreported site between the Asp and Leu residues in the middle of the unique domain. 

## 2. Results and Discussion

### 2.1. Proteolytic Processing of Matrilin-2

**Figure 1:**
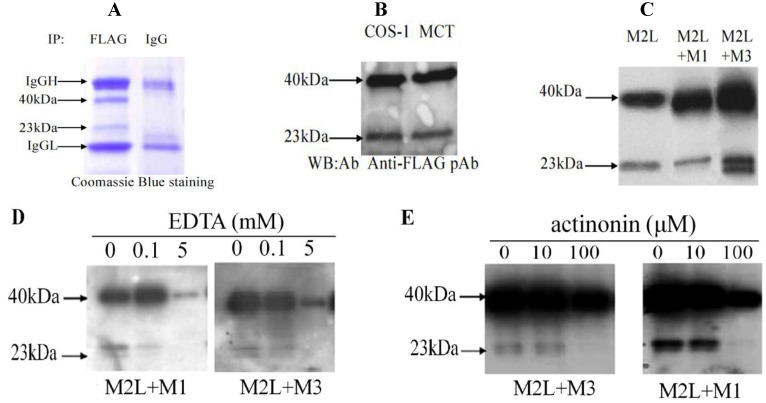
Matrilin-2 Proteolysis *in vitro*. (**A**) Purified M2L peptides visualized on a 4%–15% SDS-PAGE gradient gel. Conditioned medium from M2L transfected COS-1 cell line was affinity purified with an anti-FLAG monoclonal antibody, separated by electrophoresis and visualized by Coomassie blue staining. Mouse normal IgG indicates an control experiemnt in which IgG replaced anti-FLAG monoclonal antibody. Non-specific IgG heavy chain (IgGH) and IgG light chain (IgGL) were indicated; (**B**) Western blot analysis of M2L peptides from the conditioned medium collected from M2L transfected monkey kidney epithilial COS-1 cells and mouse chondrocyte MCT cells, respectively. Conditioned medium was subjected to electrophoresis and followed by western blot analysis with an anti-FLAG antibody; (**C**) Different proteolytic cleavage patterns of matrilin-2 from single or co-transfections of matrilins. Conditioned medium from Cos-1 cells transfected with M2L or co-transfection of M2L with either matrilin-1or matrilin-3 was analyzed by electrophoresis on an 4%–15% SDS-PAGE gel followed by western blot with an anti-FLAG antibody. Note the apperance of a doublet of cleavage products in co-transfection samples; (**D**) Western blot analysis of matrilin-2 peptides from the conditioned medium of Cos-1 cells co-transfected with M2L and matrilin-1, or with M2L and matrilin-3. Different concentrations of EDTA were added into culture medium for 72 h. The conditioned medium was collected and subjected to SDS-PAGE electrophoresis followed by western blotting using an anti-FLAG polyclonal antibody; (**E**) Actinonin inhibit matrilin-2 cleavage. Western blot analysis of matrilin-2 peptides from the conditioned medium of Cos-1 cells co-transfected with M2L and matrilin-1, or with M2L and matrilin-3. Different concentrations of actinonin were added into culture medium for 72 h. The conditioned medium was collected and subjected to SDS-PAGE electrophoresis followed by western blotting using an anti-FLAG polyclonal antibody.

To study the role of the unique domain of matrilin-2 in the proteolytic cleavage of matrilin-2, we cloned a mouse matrilin-2 cDNA (M2L) that contained the *C*-terminal half of the molecule including the vWFA2 domain, the unique domain, and the coiled-coil domain. After the M2L cDNA was transfected into Cos-1 cells, secreted recombinant MATN2 (rMATN2) peptides in the conditioned medium were affinity purified using the antibody against the FLAG tag at the N-terminus of rMATN2. In addition to the full length M2L peptide at the expected molecular weight of 40kDa, another smaller peptide at 23 kDa was detected (Figure 1A). This suggested that the 40 kDa full-length rMATN2 was cleaved to yield an *N*-terminal peptide of 23 kDa. This cleavage product was detected in both Cos-1 cells and MCT chondrocytes cultures (Figure 1B). Thus, both kidney and cartilage derived cells contain the proteolytic activity. Interestingly, expression of rMATN2 alone exhibited a different proteolytic pattern than co-expression of rMATN2 with other matrilins. While single transfection of M2L generated a 23 kDa *N*-terminal MATN2 proteolytic fragment, its co-transfection with a MATN1 or MATN3 cDNA generated an additional *N*-terminal 25 kDa matrilin-2 fragment (Figure 1C). Thus, co-expression of matrilin-2 with other matrilins affects the processing of matrilin-2. To determine whether such proteolysis involved matrix metalloproteinase, we added EDTA, a matrix metalloproteinase inhibitor (Figure 1D), and actinonin, which inhibits 100% of aggrecanase and 22.5% MMP at 100 M [33] into the medium (Figure 1E). Both compounds inhibited rMATN2 proteolysis (Figure 1D,E).

### 2.2. Matrilin-2 Was Cleaved by ADAMTS-4 and ADAMTS-5 but Not ADAMTS-1

**Figure 2:**
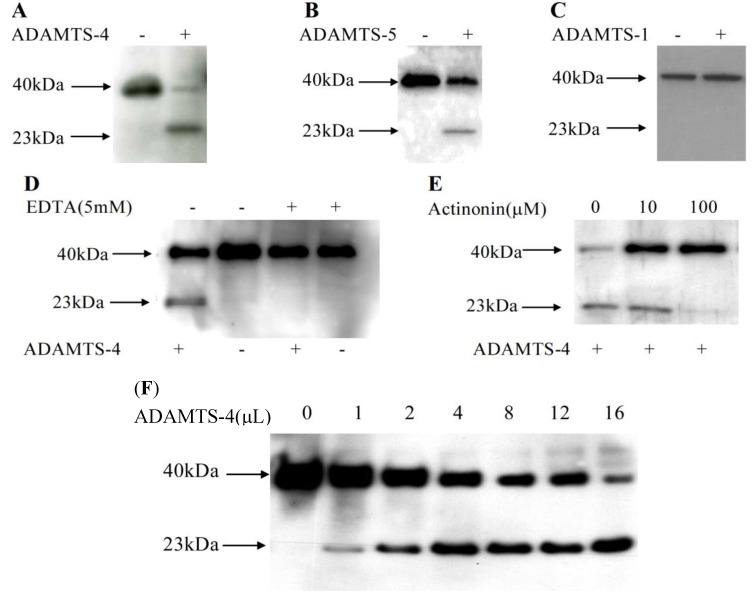
Matrilin-2 was cleaved by purified human ADAMTS-4 and ADAMTS-5 specifically. Matrilin-2 peptides were incubated with or without purified recombinant ADAMTS-4 (**A**), ADAMTS-5 (**B**) and ADAMTS-1 (**C**). Western blot analysis of the conditioned medium of Cos-1 cells was performed using an anti-FLAG polyclonal antibody. EDTA (**D**) or actinonin (**E**) was added into cell culture. These conditioned media were incubated with or without purified recombinant ADAMTS-4 followed by western blot analysis with an anti-FLAG polyclonal antibody. (**F**) ADAMTS-4 cleavage of matrilin-2 was dosage dependent. Purified M2L (1 ng) in 10 μL reaction buffer was incubated with purified human recombinant ADAMTS-4 (5 μg/25 μL) at the contents of 0, 1, 2, 4, 8, 12, and 16 μL at 37 °C for 24 h, followed by western blot analysis using anti-flag poly clonal antiboby.

To determine whether aggrecanases were responsible for proteolysis of matrilin-2, conditioned medium from M2L transfected cells was harvested and incubated with purified recombinant human ADAMTS-4 (aggrecanase-1) at 37 °C for 24 h. Incubation with ADAMTS-4 greatly increased the yield of the 23 kDa cleavage product (Figure 2A right). ADAMTS-5 incubation also significantly increased the 23 kDa rMATN2 N-terminal cleavage peptide (Figure 2B). However, ADAMTS-1 incubation did not increase the yield of matrilin-2 cleavage peptide (Figure 2C). Furthermore, ADAMTS-4 proteolysis of rMATN2 was completely inhibited by 5 mM EDTA, a matrix metalloproteinase inhibitor (Figure 2D) and by 100 μM actinonin (Figure 2E). Therefore, we concluded that aggrecanases were involved in rMATN2 proteolysis. Furthermore, western blot analyses indicated that proteolysis of matrilin-2 was dependent on the concentration of ADAMTS-4 (Figure 2F).

### 2.3. Identification of the Proteolytic Site in the Unique Domain of Matrilin-2

**Figure 3:**
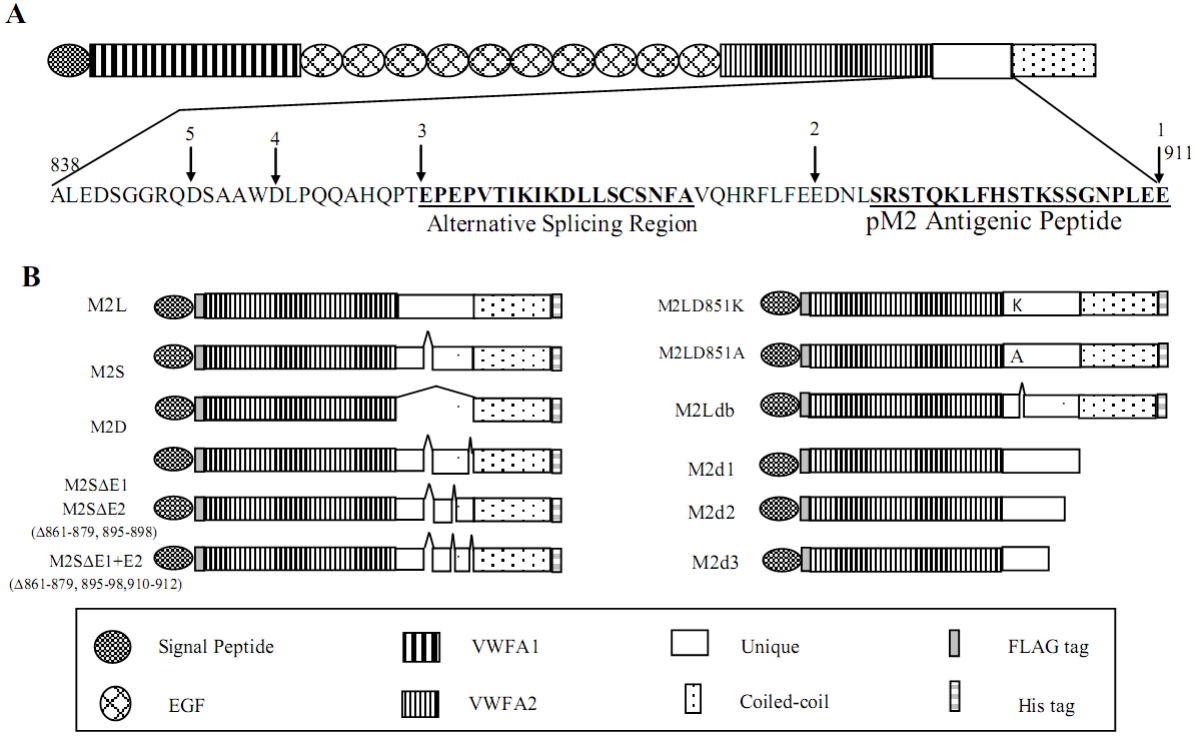
Diagram of matrilin-2 and its cDNA constructs. (**A**) A schematic diagram of matrilin-2 and the amino acid sequence of the unique domain. Alternative splicing region and the antigenic peptide for generation of anti-matrilin-2 antibody were indicated. The numbers correspond to different truncation sites within the domain. (**B**) Matrilin-2 cDNA constructs with deletions and mutations in the unique domain. M2L(Long) contains the entire unique domain (A^838^ to E^911^). M2S(Short) contains the short form of the unique domain without the alternative splicing region (E^861^ to A^879^). M2D(Deleted) has the entire unique domain deleted. M2d1, M2d2, M2d3 were matrilin-2 truncated products in which the stop codon was added after E^911^ (position 1), E^888^ (position 2), E^861^ (position 3) in M2L respectively. Position 4 between D and L is the putative unique ADAMTS cleavage site in matrilin-2, while position 1 between E^911^ and S^912^ is the second putative ADAMTS cleavage site, similar to the sites in other matrilins.

To identify the proteolytic cleavage sites within the unique domain (Figure 3A), we generated a series of mouse matrilin-2 cDNAs containing various deletions and mutations in the unique domain of matrilin-2 (Figure 3B).

**Figure 4:**
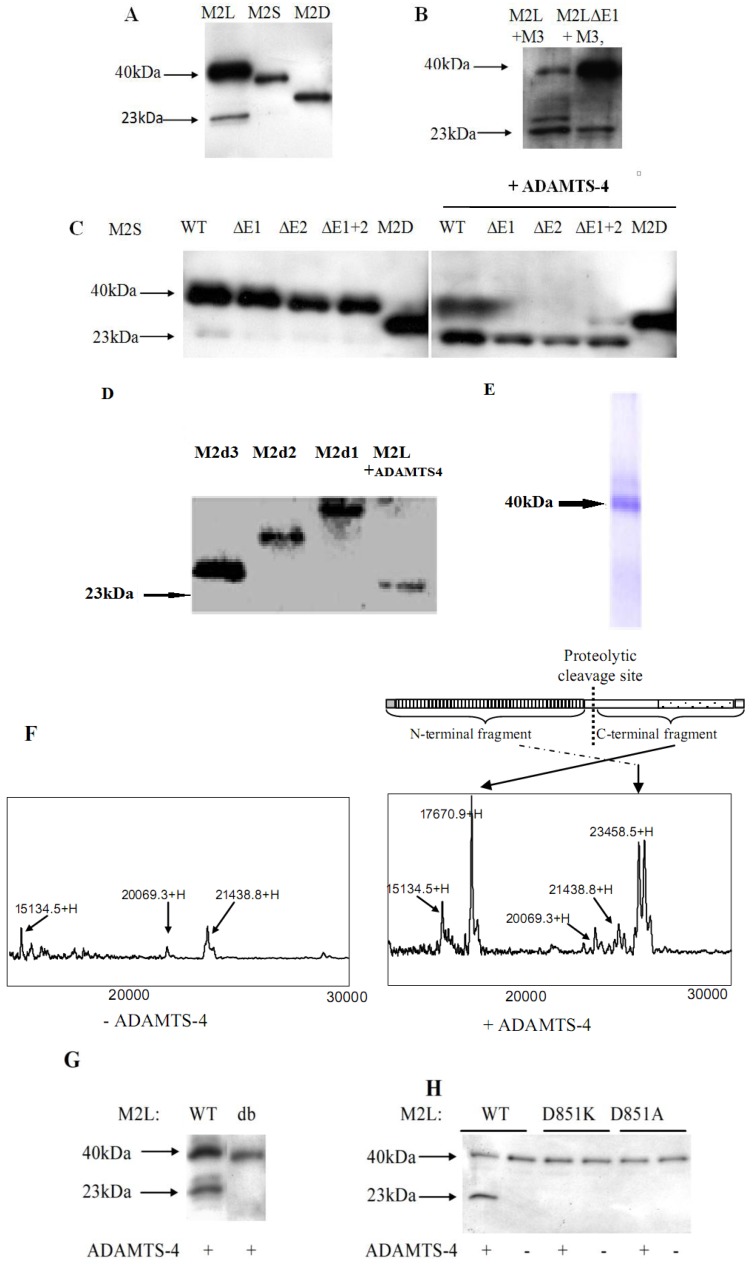
Identification of matrilin-2 proteolytic cleavage sites. **(A**) The unique domain is required for matrilin-2 proteolysis. Western blot analysis of matrilin-2 products in the conditioned medium collected from Cos-1 cells co-transfected with M2L and M1 (matrilin-1), M2S and M1, or M2D and M1 respectively, and western blot with an anti-FLAG antibody; (**B**) The EES site in the unique domain of matrilin-2 is involved in the proteolysis by ADAMTS-4. Wild type M2L and M2LΔE1 (EES deletion) were co-transfected with M3 (matrilin-3) cDNA in Cos-1 cells. Condidtioned medium was incubated with recombinant human ADAMTS-4 before western blot analysis with an anti-FLAG antibody; (**C**) ADAMTS-4 cleaves matrilin-2 mutants harboring glutamate acid deletions in the unique domain, including M2S (WT), M2SΔE1, M2SΔE2, and M2SΔE1+2. M2D was used as a negative control. Western blot analysis was performed with the conditioned medium collected from Cos-1 cells after treatment with recombinant human ADAMTS-4; (**D**) Western blot analysis of matrilin-2 truncation products M2d1, M2d2 and M2d3 in comparison to the 23 kDa M2L ADAMTS-4 cleavage product; (**E**) Recombinant M2L was purified from the conditioned medium of M2L transfected Cos-1 cells by Ni-NTA procedure and visualized by Coomassie blue staining; (**F**) Mass spectrometry analysis of purified M2L product incubated with or without recombinant human ADAMTS-4. Molecular mass of M2L cleavage product is indicated; (**G**) and (**H**) Matrilin-2 cleavage site mutants are resistant to ADAMTS-4 proteolysis. Wild-type matrilin-2, site deletion mutant (db) or point mutation (D851K, D851A) mutant were incubated with purified recombinant human ADAMTS-4 followed by western blot analysis with anti-FLAG polyclonal antiboby.

To determine whether the proteolytic cleavage sites were located in the unique domain, we transfected M2L, M2S, and M2D into Cos-1 cells. While transfection of M2L that contained the entire unique domain resulted in the 23 kD cleavage product, transfection of M2S with a 19 amino acid deletion in the unique domain produced a low level of cleavage product. Transfection of M2D, which lacked the entire unique domain, produced no cleavage product (Figure 4A). These results suggested that the cleavage sites were located within the unique domain.

Because the conserved ADAMTS cleavage site in aggrecan, bevican, and matrilin-3 occurred after the glutamic acid residue, we systematically deleted glutamic acid residues within the unique domain. While the wild type matrilin-2 co-transfection with matrilin-3 produced two cleavage products at 23 and 25 kDa, the M2LΔE1 mutant which lacked the glutamic acid-glutamic acid-aspartic acid residues at position 910–912, produced only the 23 kDa product (Figure 4B), suggesting that these three amino acid residues were required for the ADAMTS generation of the 25 kDa, but not the 23 kDa cleavage peptide. Furthermore, ADAMTS-4 incubation produced a 23 kDa cleavage peptide in the MATN2 mutants lacking one or both glutamic acids (E1 or E2). This suggested the existence of a second (previously unreported) ADAMTS-4 cleavage site in the unique domain, and one that did not follow a glutamic acid residue.

To locate this novel ADAMTS-4 cleavage site, we created a set of truncated matrilin-2 constructs by adding the stop codon at three specific sites within the unique domain (Figure 3B). M2d1 truncated the protein at the *C*-terminal end of the unique domain. M2d2 truncated the protein at the C-terminus of the alternative splicing region. And M2d3 truncated the protein at the N-terminus of the alternative-splicing region. Western blot analyses indicated that the ADAMTS-4 cleavage product was smaller than all three truncated products (Figure 4D), suggesting that the cleavage site was located within the first 25 amino acids of the N-terminal region before the alternative splicing region in the unique domain.

To more precisely identify the cleavage site, we affinity-purified the full-length 40 kDa rMATN2 from the conditioned medium (Figure 4E). Incubation of purified rMATN2 with recombinant ADAMTS-4 at 37 °C for 24 h resulted in two peaks by SELDI mass spectrometry, representing matrilin-2 peptides of 17,670.9 Dalton and 23,658.5 Dalton (Figure 4F). Based on the calculated molecular weights, we calculated the matrilin-2 proteolytic cleavage site to be between the D^851^ and L^852^ residues (Figure 4D). To further confirm the matrilin-2 cleavage site, we performed deletion and point mutation analyses of the putative cleavage site. While ADAMTS-4 could cleave the wild type rMATN2, it could not cleave the mutant M2Ldb (∆W^850^DLP^853^) in which the cleavage site was deleted (Figure 4G). Furthermore, ADAMTS-4 could not cleave the matrilin-2 peptides harboring point mutations of the putative cleavage site, including M2LD851A (in which changed the negatively charged amino acid aspartic acid had been changed to the non-charged valine) and M2LD851K (in which the negatively charged amino acid aspartic acid had been changed to the positively charged amino acid lysine) (Figure 4G). In contrast, the point mutations of glutamic acid residues in the middle of the unique domain did not change the matrilin-2 cleavage pattern (Figure 4C).

Of the two cleavage sites, one follows a glutamic acid residue and the other does not. A glutamic acid residue commonly precedes the cleavage sites of many glycosaminoglycans (GAG) proteins. For example, ADAMTS-4 and ADAMTS-5 cleave the following substrates after the glutamic acid residue: Aggrecan (Glu^373^/Ala^374^, Glu^1545^/Gly^1546^, Glu^1714^/Gly^1715^, Glu^1819^/Ala^1820^, Glu^1919^/Leu^1920^) [15,19,34], versican (Glu^441^/Ala^442^) [35], and brevican (Glu^395^/Ser^396^) [36]. Less commonly, ADAMTS-4 and ADAMTS-5 also cleave substrates at other sites. For example, ADAMTS-4 and ADAMTS-5 cleave α_2_-macroglobulin at the Met^690^/Gly^691^ [37] and aggrecan at Asn^341^/Phe^342^ [38].

ADAMTS-4 and ADAMTS-5 cleave not only matrilin-2 but also other members of the matrilin family. In addition, Matrilin-4 has been reported to be cleaved at a site between the vWFA2 domain and the coiled-coil domain [7], similar to the location of the proteolytic site in matrilin-2 identified in this study. Matrilin-1 also possesses a proteolytic cleavage site between the glutamic acid and glycine residues in the hinge region [29]. All four matrilin members possess a proteolytic cleavage site after glutamic acid (matrilin-3 at Glu^435^/Ala^436^ [30], and matrilin-4 at Glu^570^ Glu^571^/G^572^) [31]. However, matrilin-2 may be unique in that it possesses two proteolytic cleavage sites, one of which follows a glutamic acid residue and the other of which follows an aspartic acid residue.

### 2.4. Matrilin-2 Proteolytic Cleavage in Vivo and the Proteolytic Susceptibility of the Splicing Variants

**Figure 5:**
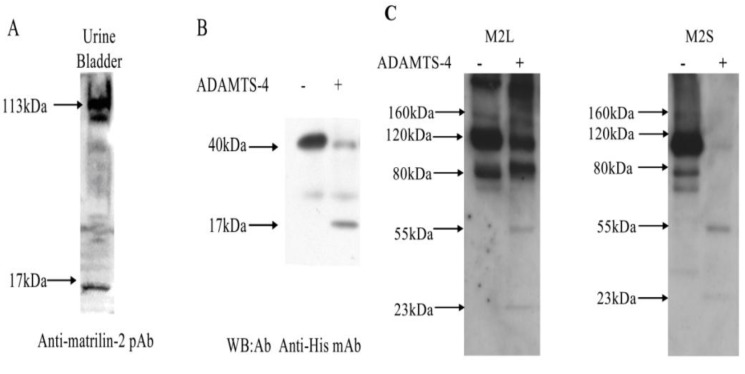
Matrilin-2 proteolytic cleavage *in vivo* and the proteolysis susceptibility of the splicing variants. (**A**) Matrilin-2 proteolytic cleavage product in mouse urine bladder. Urine bladder from newborn mice was homogenized and analyzed by electrophoresis in a 4%–15% SDS-PAGE gel under reducing conditions, followed by western blot analysis using a polyclonal antibody pM2 against matrilin-2. A full length 113 kDa matrilin-2 monomer and the 17 kDa *C*-terminal cleavage product were indicated; (**B**) Western blot analysis of *C*-terminal matrilin-2 proteolytic cleavage product. M2L transfected cells were incubated with ADAMTS-4 for 24 h. Electrophoresis was performed in a 4%–15% SDS-PAGE gel under reduced conditions, followed by western blot analysis using a monoclonal antibody against the His tag at the C-terminus; (**C**) M2L or M2S transfected cells were incubated with ADAMTS-4 for 24 h. Electrophoresis was performed in a 4%–15% SDS-PAGE gel under non-reduced conditions, followed by western blot analysis using a monoclonal antibody against the FLAG tag at the N-terminus.

To determine whether the processing also occurred *in vivo*, we generated a polyclonal antibody (pM2) against a synthetic peptide from the unique domain of mouse matrilin-2 (Figure 3A). Since this antigenic peptide is located at the *C*-terminal side to the D^851^/L^852^ cleavage site, we reasoned that this antibody should detect a 17 kDa (the difference in size between the 40 kDa full length matrilin protein and the 23 kDa N-terminal fragment) *C*-terminal MATN-2 proteolytic product resulted from matrilin-2 proteolysis. We analyzed tissue extracts from the mouse urine bladder that were known to express matrilin-2 [39] with western blot using the pM2 antibody. A 17 kDa matrilin-2 proteolytic fragment was indeed detected in addition to the full-length MATN2 from tissue extracts (Figure 5A). Thus, we concluded that proteolytic processing of MATN2 also occurred *in vivo*. Using an antibody against the His-tag at the C terminal end of rMATN2, we detected a 17kDa product with the recombinant MATN2 (Figure 5B), suggesting that the *in vivo* and *in vitro* processing of matrilin-2 involved the same proteolytic site.

The long and short splice variants of matrilin-2 mRNA were expressed in all mouse skeletal and non-skeletal tissues [32]. To determine the susceptibility of the MATN2 splice variant peptide products to ADAMTS-4 proteolysis, we incubated the protein products of M2L and M2S with ADAMTS-4 (Figure 5C). Prior to ADAMTS-4 proteolysis, M2L protein products existed as dimers (80 kDa), trimers (120 kDa), tetramers (160 kDa), or multimers, and M2S protein products existed as dimers (74 kDa), trimers(111 kDa), tetramers (148 kDa), or multimers. After ADAMTS-4 proteolysis, the N-terminal cleavage peptide of 23 kDa appeared in both splicing variant peptide products as well as with the full length peptide linked with the *C*-terminal cleavage peptide (57 kDa (= 40 + 17) in M2L, and 54 kDa (= 37 + 17) in M2S) (Figure 5C). Most strikingly, M2S was much more extensively cleaved than M2L, suggesting the long splice variant peptide product of MATN2 was more resistant to ADAMTS-4 proteolysis than the short splicing variant product.

## 3. Experimental

### 3.1. Cloning and Construction of Matrilin-2 cDNAs

Mouse matrilin-2 cDNA was cloned by RT-PCR from RNA isolated from sternal cartilage of newborn mice. Total RNA was isolated using the RNeasy kit (Qiagen, GmbH, Hilden, Germany). RT-PCR of matrilin-2 mRNA was performed using Titan One Tube RT-PCR system (Boehringer Mannheim, Indianapolis, IN, USA) according to manufacturer’s instructions. Two step-PCR was used in the same tube under the following conditions: 94 °C for 30 s, 50 °C for 30 s, and 68 °C for 1.5 min for 10 cycles. Then, the annealing temperature was raised to 55 °C for another 20 cycles. The matrilin-2 cDNA as well as cDNAs encoding chicken matrilin-1 and -3 from previous studies [28,40] were cloned into an expression vector pcDNA3.1/V5-His (Invitrogen, Carlsbad, CA, USA). Genetic engineering including the addition of a N-terminus FLAG tag, truncated mutations (M2Ld1, M2Ld2, M2Ld3), deletion mutations (M2SΔE1, M2SΔE2, M2SΔE1+E2, M2Ldb), and point mutations (D851A, D851K) within the unique domain (Figure 1) was performed by overlapping PCR with the described primer sets (Table 1). The modified cDNAs were cloned between the Xho I and Not I restriction sites of pcDNA3.1. All constructs were confirmed by DNA sequencing.

**Table 1:** A list of primers for generating matrilin-2 cDNA constructs.

Primers	Primer Sequences (5'–>3')	PCR Purpose
T7	T AATACGACTCACTATAGGG	Cloning into pcDNA3.1/V5-His
BGH	CTAGAAGGCAACAGTCGAGG	
Mat2flgas	GCATCTTTTGTCATCATCGTCCTT	Adding a FLAG tag in matrilin-2
Mat2flgs	GATGACAAAAAGAGATGCACTGAAGGC	
M2d1R	CGCTCGAGTCATTCTTCCAAAGGGTTCC	Creating M2d1 deletion mutant
M2d2R	CGCTCGAGTCATTCTTCAAACAGGAATC	Creating M2d2 deletion mutant
M2d3R	CGCTCGAGTCATTCTGTTGGCTGGTGGC	Creating M2d3 deletion mutant
M2Ldb2F	GACTCAGCAGCACAGCAGGCCCACCA	Creating M2Ldb mutant deleted 850WDLP853
M2Ldb2R	GTGGGCCTGCTGTGCTGCTGAGTCCT	
M2LD851KF	AGCATGGAAAGCTGCCACA	Creating M2LD851K mutant
M2LD851KR	TGTGGCAGCTTCCATGCT	
M2LD851AF	AGCATGGGCCCTGCCACAGCAG	Creating M2LD851A mutant
M2LD851AR	CCTGCTGTGGCAGGGCCCATGCT	
DETA3F	TTCCTGTTTAATCTTTCACGGTCTACACA	M2SΔE2 (ΔGlu887 GluAsp889), M2SΔE1 + E2(ΔGlu887 GluAsp889, ΔGlu910 GluSer912)
DETA3R	GAAAAGTTAAACAGGAATCTATGTTGCA	
DETA4F	CCCTTTGCAGGACCAATGCAAATGTGA	Creating M2SΔE1 (ΔGlu910 GluSer912), M2SΔE1 + E2 (ΔGlu887 GluAsp889, ΔGlu910 GluSer912)
DETA4R	GTCCTGCAAAGGGTTTCCTGAAGATTT	

### 3.2. Transfection of Matrilin cDNAs

cDNA constructs of matrilin-3 and -1 were transfected into COS-1 cells or MCT chondrocytes [41] using LIPOFECTAMINE 2000 (Life Technology, Rockville, MD, USA) according to the manufacturer’s instructions. Briefly, COS-1 cells or MCT chondrocytes were trypsinized and counted. Each 60 mm plate was seeded with 6 × 10^5^ cells. They were incubated at 37 degrees C overnight in DMEM (Gibco, Life Technologies, Grand Island, NY, USA) supplied with 10% FBS (Life technology) to allow the cells to reach 70% confluence. The following day, the cells were rinsed with DMEM and subjected to a DNA/LIPOFECTAMINE 2000 (Life Technology) mix for 5 h. For single transfections, 5 μg of DNA were used. For co-transfections, 4 μg DNA of each of the constructs were used. The DNA/LIPOFECTAMINE 2000 mixture was aspirated and replaced with 3 mL DMEM supplied with 1% FBS. The media from transfected cell culture was collected at 72 h after transfection. Cells were lysed on ice for 10 minin a lysis buffer as previously described [28]. Cell lysates were centrifuged at 4 °C for 10 min. Supernatant of the cell lysate, as well as of the conditioned medium, was analyzed using western blots. Some transfected cells were treated with EDTA, or actinonin at indicated concentrations for 48 h before the collection of the conditioned medium.

### 3.3. SDS-Polyacrylamide Gel Electrophoresis and Western Blot

Western blot analysis was performed with collected conditioned medium, with cell lysates from transfected cell cultures, or with urine bladder tissue extracts from new born mice as described previously [42]. Equal amounts of protein were loaded in each lane after quantification of the protein in the medium samples by bicinchoninic acid assay (BCA assay). For non-reducing conditions, collected samples were mixed with standard 2× SDS gel-loading buffer [28]. For reducing conditions, the loading buffer contains 5% β-mercaptoethanol and 0.05 M DTT. Samples were boiled for 10 min before being loaded onto 10% SDS-PAGE gels or 4%–20% gradient gels. After electrophoresis, proteins were transferred onto Immobilon-PVDF membrane (Millipore Corp., Bedford, MA, USA) in 6 mM Tris, 192 mM glycine, and 15% methanol. The membranes were blocked in 2% bovine serum albumin fraction V (Sigma Co., St. Louis, MO, USA) in PBS for 30 min and then probed with antibodies.

The primary antibodies included a monoclonal antibody against the His tag (diluted 1:5000) (Invitrogen) and a monoclonal antibody against FLAG (diluted 1:1000) (Affinity BioReagents, Golden, CO, US). To generate a polyclonal antibody against matrilin-2, a peptide encoding 20 amino acids (SRSTQKLFHSTKSSGNPLEE) from the unique domain of mouse matrilin-2 was synthesized. The antibody was raised by immunizing rabbits with the synthetic peptide followed by affinity purification. Horseradish peroxidase conjugated goat anti-mouse or goat anti-rabbit IgG (H + L) (Bio-Rad Laboratories, Melville, NY, USA), diluted 1:3000, was used as a secondary antibody. Visualization of immunoreactive proteins was achieved using the ECL Western blotting detection reagents (Amersham Corp., Heights, IL, USA) and exposing the membrane to Kodak X-Omat AR film. Molecular weights of the immunoreactive proteins were determined against two different sets of protein marker ladders.

### 3.4. In Vitro Analysis of Matrilin-2 Proteolysis

12 μL conditioned medium from matrilin-2 transfected cell culture was incubated with 0.8 μL purified recombinant human ADAMTS-4 (Phe213-Pro431) (Calbiochem, San Diego, CA, US) (final concentration: 0.013 μg/μL), recombinant human ADAMTS1 (R&D system, 2197-AD-020) (final concentration: 0.015 μg/μL) or 1 μL purified recombinant human ADAMTS-5 (CHEMICON, Temecula, CA, USA) (final concentration: 0.015 μg/μL), in a reaction buffer (50 mM Tris, 100 mM NaCl, 10 mM CaCl_2_, pH 7.5) for 24 h at 37 °C. Recombinant matrilin-2 was also purified from conditioned medium of cells transfected with matrilin-2 cDNA using Ni-NTA agarose (Qiagen, GmbH, Hilden, Germany). 5 μL of purified MATN2 recombinant protein was incubated with 2 μL purified recombinant ADAMTS-4 (Calbiochem) at 37 °C for 24 h. The incubation mixture was separated by SDS-PAGE electrophoresis and analyzed by western blot using the anti-FLAG polyclonal antibody and the anti-His monoclonal antibody. The incubation mixture was also analyzed by SELDI mass spectrometry as described previously [43].

## 4. Conclusions

The unique domain of matrilin-2 connects the *C*-terminal coiled-coil oligomerization domain with the protein binding VWA domain arms of the bouquet-shaped molecule. We have identified two proteolytic sites within the unique domain in matrilin-2. The cleavage products were present both *in vitro* and *in vivo*. We demonstrated that ADAMTS-4 (aggrecanase-1) and ADAMTS-5 (aggrecanase-2), but not ADAMTS-1, were capable of cleaving matrilin-2. ADAMTS-4 cleaved matrilin-2 not only at the N-terminus of the coiled-coil domain (as in other matrilin proteolytic sites), but also at a previously unreported site between Asp^851^ and Leu^852^.
